# Integrative Insights into the Immunopathogenesis and Organ-Specific Immunological Mechanisms of Long COVID: A Narrative Review

**DOI:** 10.3390/v18040458

**Published:** 2026-04-10

**Authors:** Supriya Mahajan, Saurabh Mahajan, Nidhi Kaushik

**Affiliations:** 1 Department of Microbiology, School of Medical Sciences and Research, Sharda University, Greater Noida 201306, Uttar Pradesh, India; 2 Department of Neurosurgery, Numed Super Speciality Hospital, Greater Noida 201306, Uttar Pradesh, India

**Keywords:** Long COVID, SARS-CoV-2, immunopathogenesis

## Abstract

Long COVID (LC), also referred to as post-acute sequelae of SARS-CoV-2 infection, is characterized by persistent symptoms originating 3 months following acute COVID-19, lasting for at least two months and frequently affecting individuals who initially experienced mild to moderate disease. The clinical spectrum is heterogeneous, involving respiratory, cardiovascular, neurological, renal, gastrointestinal, and endocrine systems, thereby posing substantial diagnostic and therapeutic challenges. Despite extensive investigation, the precise immunopathogenic mechanisms underlying LC remain incompletely defined. Accumulating evidence suggests that LC is driven by a multifactorial interplay of persistent viral antigen reservoirs, chronic immune activation, dysregulated innate and adaptive immune responses, autoimmunity, endothelial dysfunction, microvascular injury, and aberrant tissue repair. These systemic immune perturbations manifest variably across different organs, contributing to the diverse clinical phenotypes observed. However, mechanistic clarity is hindered by heterogeneity in study designs, limited longitudinal data, and the absence of standardized immunological profiling. This narrative review provides integrative insights into the immunopathogenesis of LC, synthesizing current evidence on systemic immune dysregulation and organ-specific immunological mechanisms. A conceptual framework is proposed to facilitate a structured understanding of this complex syndrome and to guide future research toward targeted immunomodulatory strategies.

## 1. What Is Long COVID?

Long COVID (LC) is defined as a post-COVID-19 condition that occurs in individuals with a history of probable or confirmed SARS-CoV-2 infection, usually 3 months from the onset of COVID-19, with symptoms that last for at least 2 months and cannot be explained by an alternative diagnosis [1]. Although attempts were made to re-designate it with other terms such as PASC (post-acute sequelae of SARS-CoV-2 infection), the patient-led term LC was persuasively argued for and finally retained [2]. About 200 symptoms have been described so far, involving multiple organ systems [3], with 10–40% of people infected with COVID-19 showing prolonged symptoms [4]. Numerous diverse immunological mechanisms have been hypothesized linking SARS-CoV-2 infection to the diverse array of persistent symptoms, which are described below.

## 2. Methods

### 2.1. Literature Search Strategy

A comprehensive literature search was conducted to identify relevant studies addressing the immunopathogenesis and organ-specific immune mechanisms of LC. Electronic databases, including PubMed/MEDLINE, Scopus, and Web of Science, were searched for articles published from January 2020 to March 2026.

The search strategy combined Medical Subject Headings (MeSHs) and free-text terms related to LC and immune mechanisms. Key search terms included combinations of:

- “Long COVID” OR “post-acute COVID-19 syndrome” OR “PASC”;
- “SARS-CoV-2”;
- “immune dysregulation” OR “immunopathogenesis” OR “immune response”;
- “autoimmunity”, “viral persistence”, “chronic inflammation”, “endothelial dysfunction”, “microvascular injury”;
- “organ-specific”, “neurological”, “cardiovascular”, “pulmonary”, “renal”, “gastrointestinal”, “endocrine”.

Reference lists of relevant articles and key reviews were also manually screened to identify additional eligible publications.

### 2.2. Study Eligibility and Selection

This narrative review aimed to synthesize current knowledge rather than perform a formal systematic review. Studies were selected based on relevance to the immunological mechanisms of LC. The following inclusion criteria were applied:

- Peer-reviewed original research articles, systematic reviews, meta-analyses, and high-quality narrative reviews;
- Human studies and relevant experimental or translational research providing mechanistic insights;
- Articles focusing on immune dysregulation, viral persistence, autoimmunity, inflammation, endothelial dysfunction, and organ-specific manifestations of LC;
- Publications written in English.

The following were excluded:

- Case reports with limited mechanistic insight;
- Non-scientific commentary or opinion pieces without supporting evidence;
- Studies unrelated to LC immunology.

### 2.3. Data Synthesis

Eligible studies were critically evaluated and integrated into a thematic narrative synthesis. Evidence was organized into key mechanistic domains, including immune dysregulation, persistent viral reservoirs, autoimmunity, immunothrombosis, and organ-specific immune pathways. Emphasis was placed on identifying areas of consensus, conflicting findings, and remaining knowledge gaps to provide an integrative conceptual framework for LC pathogenesis.

## 3. Immunological Mechanisms of LC

### 3.1. T-Cell and B-Cell Dysregulation

Studies have demonstrated that resolution of initial SARS-CoV-2 infection led to a prolonged period of abnormal T-cell activation [5]; increased CD4+ T-cells; exhausted SARS-CoV-2-specific CD8+ T-cells with increased expression of T-cell exhaustion markers PD-L1 and TIGIT [6], which has been most marked in older individuals due to age-related immunosenescence; and/or poorer T-cell cross-reactivity between different coronaviruses [7]. Similar results were shown with B-cells, resulting in increased activated B-cells [8] and exhausted B-cells [9]. Loss of T-cell function has been hypothesized to be due to the large number of viral-specific T-cells that are produced during acute SARS-CoV-2 infection, especially in those with prolonged viral shedding [10]. Studies have also demonstrated increased production of type I and III interferons in LC which can be explained by the fact that dysfunctional interferon responses played a key role in the development of severe acute COVID-19 [11,12]. Another risk factor that increases the probability of patients developing LC is the presence of lower concentrations of specific immunoglobulin (Ig)-neutralizing antibodies IgM and IgG3 [13], along with miscoordination between T-cell and B-cell responses leading to persistently elevated altered patterns of immunoglobulins, which reflects ongoing immune stimulation even when the virus is no longer detectable [14].

### 3.2. Viral Persistence as an LC Driver

The literature evidence increasingly supports viral persistence as a contributing mechanism in LC, with viral RNA, proteins, and in some cases intact virus detected across multiple organs, including the brain, gut, lungs, heart, lymphoid tissues, and endocrine organs [15]. This persistence may arise from delayed or impaired immune signaling, exaggerated and exhausted T-cell responses, and incomplete viral clearance [16]. PCR or antigen detection from biopsy or autopsy tissue samples and stool and blood samples have shown evidence of viral reservoirs, with the gastrointestinal tract emerging as a key potential reservoir because of its accessibility and high ACE2 expression [1,17,18,19,20]. Importantly, viral presence in intestinal biopsies has been associated with ongoing symptoms even when stool testing is negative, indicating that fecal PCR alone may underestimate gut persistence [21]. Circulating spike protein (S) has also been identified in a substantial proportion of LC patients up to one year after infection and correlates with multisystem involvement [15]. Autopsy studies further demonstrate widespread viral distribution and prolonged RNA detection in diverse tissues, including the brain, months after infection [22]. Continued antigen exposure likely maintains activation of both innate and adaptive immune responses, while T-cell exhaustion reduces the efficiency of viral clearance, creating a self-perpetuating cycle of immune stimulation and inflammation [23,24]. Collectively, these findings suggest that persistent viral reservoirs may drive chronic immune activation and contribute to the heterogeneous clinical manifestations of LC depending on the affected tissues [23].

### 3.3. Release of Cytokines in LC Patients

Acute COVID-19, whether mild or severe, has consistently been associated with a pronounced hyperinflammatory state marked by increased circulating cytokines, particularly IL-6, IL-8, and TNF-α [25]. Similar immune alterations have been reported in LC, where IL-6, IL-1β and TNF-α remain persistently elevated [26]. Evidence further indicates that individuals with LC exhibit sustained activation of innate immune cells, reduced pools of naïve T and B lymphocytes, and prolonged upregulation of type I (IFN-β) and type III (IFN-λ1) interferons that can persist for up to eight months following the initial infection [27]. Meta-analytic findings also support the persistence of inflammatory mediators, including IL-6, MCP-1/CCL2, TNF-α, and IFN-γ, well beyond the acute phase, potentially underlying ongoing symptoms such as fatigue, malaise, and cognitive dysfunction (“brain fog”) [28]. In addition, sustained cytokine activity may disturb neural immune balance and increase blood–brain barrier permeability, thereby promoting chronic neuroinflammation and contributing to neurological manifestations including fatigue, mood disturbances, cognitive impairment, and brain fog [29,30]. Dysregulation of the Hypothalamic–Pituitary–Adrenal (HPA) axis plays an important role in multisystem manifestations of LC. The HPA axis regulates the body’s response to stress, wherein the hypothalamus secretes corticotropin-releasing hormone (CRH) that stimulates the pituitary to release adrenocorticotropic hormone (ACTH), which in turn induces cortisol secretion from the adrenal cortex. Proinflammatory cytokines such as IL-1, IL-6, and TNF-α can activate the HPA axis, and the cortisol produced exerts negative feedback on both the hypothalamus and pituitary while also suppressing excessive inflammation, thereby maintaining homeostasis [31]. Persistent cytokine elevation in LC leads to dysregulation of this axis, impairing cortisol-mediated negative feedback and resulting in persistent inflammation and multisystem symptoms [32]. Hence, elevated cytokines can alter neurotransmission, endocrine signaling (HPA axis), fever regulation, and metabolic processes, contributing to the multisystem features seen in LC [29]. Multiple checkpoint regulation sites with feedback inhibition are usually involved in the normal cytokine signaling pathways, allowing the cells to return to a quiescent, noninflamed state. Dysregulation of cytokine signaling has been proposed to occur in LC patients, leading to autoinflammatory disorders [33].

### 3.4. Reactivation of Latent Infections, Predominantly Epstein–Barr Virus (EBV)

**Figure 1:**
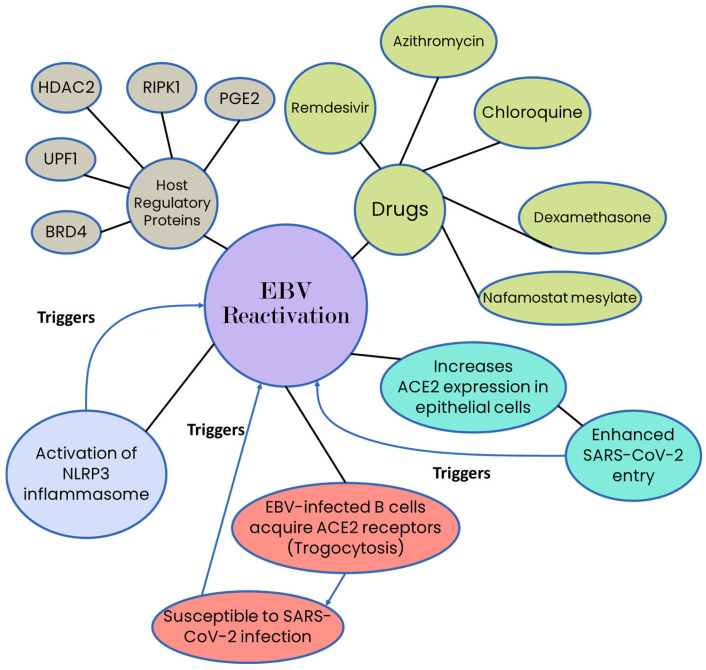
Factors associated with EBV reactivation.

EBV reactivation is common in LC patients, with reported rates ranging from ~13% to as high as 65%, particularly in severe and ICU cases. EBV reactivation is consistently associated with greater disease severity, higher inflammatory markers (CRP, D-dimer), lymphocyte dysfunction, longer ICU stay, and increased complications, including secondary infections and mortality. Several drugs used during COVID-19 management, such as remdesivir, azithromycin, chloroquine, dexamethasone, and nafamostat mesylate, as well as interactions of SARS-CoV-2 proteins with host regulatory proteins (e.g., BRD4-, UPF1-, HDAC2-, RIPK1-, and PGE2-associated pathways), can unintentionally trigger EBV lytic reactivation. EBV lytic replication increases ACE2 expression in infected epithelial cells via EBV transcriptional activators, resulting in enhanced SARS-CoV-2 entry and creating a bidirectional amplification loop between the two viruses [34]. Also, SARS-CoV-2 is known to cause activation of the NLRP3 inflammasome [35,36], and EBV can be reactivated by activation of the NLRP3 inflammasome [37]. Human primary B lymphocytes serve as the principal reservoirs for EBV and are infected through the interaction of the viral outer-envelope glycoprotein gp350/220 with the CD21 receptor on the B-cell surface, in conjunction with gp42 [38]. However, SARS-CoV-2 primarily utilizes the ACE2 receptor for cellular entry, which is minimally expressed on B-cells, creating an apparent paradox [39]. This paradox may be explained by trogocytosis, a biological process in which cells exchange membrane fragments and membrane-associated proteins during cell–cell contact, allowing EBV-infected B-cells to acquire ACE2 receptors and thereby potentially become susceptible to SARS-CoV-2 infection [34,40]. An alternate mechanism exhibited by the antigens of SARS-CoV-2 for entering B-cells might be exosomes secreted by cells that are infected by the virus [34,41]. It has been further speculated that reactivated EBV makes SARS-CoV-2 infections worse because it increases the expression of the ACE2 receptor [42] and also enhances the entry of SARS-CoV-2 into cells, potentially increasing viral load and symptom severity [43]. Another interesting feature is that LC patients who reported pre-existing autoimmune diseases like thyroiditis and have experienced fatigue showed higher levels of Early Antigen–Diffuse (EA-D) IgG [44]. Factors associated with EBV lytic reactivation are shown in Figure 1.

### 3.5. Autoimmunity

COVID-19 patients showed dramatically increased autoantibodies as compared to non-infected individuals [45]. Autoantibodies linked with systemic lupus erythematosus (SLE) and inflammatory myopathies are significantly higher in some LC patients and correlate with symptom severity, with no decrease in the autoantibody titers even after subsequent vaccination/booster [46]. Similarly, autoantibodies have been implicated in the pathogenesis of LC, like antibodies against angiotensin, ACE, and muscarinic receptors [8,47,48], but there are conflicting reports in the published literature regarding the association of autoimmunity with LC. Autoantibodies such as neutralizing anti-interferon antibodies, generated in acute infection, may theoretically promote the development of viral persistence and hence contribute to the development of LC; however, although detection of these antibodies in the acute phase has been associated with persistent respiratory symptoms [49], there has been a low prevalence of anti-interferon antibodies in the post-acute period [50].

Autoimmunity in LC may be triggered by a phenomenon called molecular mimicry, where a high degree of structural homology between spike protein and the human proteome has been implicated in cross-reactive autoantibody development [51]. The viral triggering of autoimmunity is intimately associated with cytokines [52] and epigenetic alterations of gene expression [53], and continuing cytokine expression can produce a low-grade chronic inflammatory state, which can further lead to activated, dysregulated microglia and neuroinflammation for LC patients [54].

### 3.6. Association with an Abnormal Antiviral Immune Response During Acute Infection

An effective early antiviral immune response is essential for the rapid clearance of SARS-CoV-2 and restoration of immune homeostasis. In a subset of individuals, however, dysregulated antiviral immunity during acute infection predisposes to persistent immune activation, tissue injury, and chronic symptoms characteristic of LC. Early induction of type I (IFN-α/β) and type III (IFN-λ) interferons is critical for restricting viral replication. Studies have demonstrated that patients who later develop LC exhibit delayed, blunted, or functionally impaired interferon responses during acute COVID-19, thus exposing them to higher viral burden and prolonged antigen exposure [55]. Abnormal antiviral response is often characterized by incomplete viral clearance, allowing viral RNA, proteins, or replication-competent virus to persist in immune-privileged sites (e.g., gut, CNS) [23], along with exaggerated production of proinflammatory cytokines (IL-6, TNF-α, IL-1β) and chemokines causing immune dysregulation, thus laying the foundation for chronic inflammation observed in LC [29].

Different studies have demonstrated divergent results in regard to immunity to SARS-CoV-2 antigens, especially S and N, which may be equal, reduced, or enhanced relative to those without persistent symptoms [1]. Su Y et al. found raised anti-N antibodies preferentially in those progressing to predominantly neurological symptoms [49]. In contrast, another study found an inverse correlation between initial anti-N antibody response during acute infection and likelihood of symptoms at 3 months or beyond, supporting the view that an inadequate initial response may predispose to LC [56]. García-Abellán J et al. found that the LC group showed significantly lower antibody levels to S, with no difference in T-cell responses [57]. Littlefield KM et al. demonstrated that pulmonary LC patients showed quite substantially enhanced CD4+ and CD8+ T-cell responses to peptide pools from S, N and membrane (M) proteins [58].

### 3.7. Mechanisms of Endothelial Dysfunction and Immunothrombosis in LC Patients

**Figure 2:**
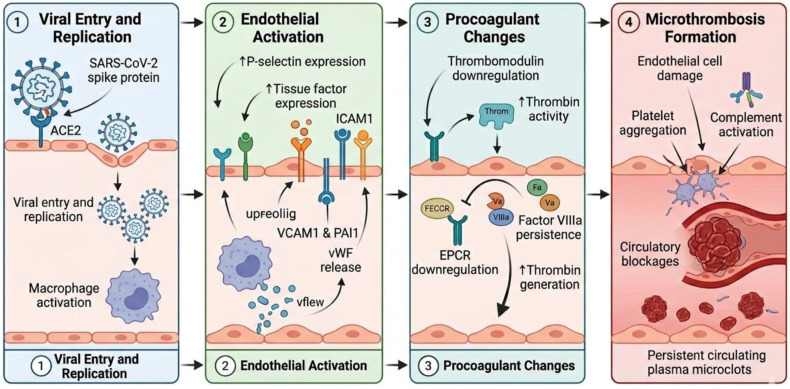
Mechanism of endothelial dysfunction and immunothrombosis in Long COVID patients.

During acute COVID-19, SARS-CoV-2 enters the respiratory epithelium via ACE2, which is followed by viral replication, activation of macrophages and complement activation, leading to endothelial cell (EC) activation. This promotes endothelial expression of proteins, such as P-selectin and tissue factor (TF); cell adhesion molecules, such as ICAM1, VCAM1 and plasminogen activator inhibitor 1 (PAI1); and the release of ultra large von Willebrand factor (vWF) multimers, along with other procoagulant changes, including downregulation of thrombomodulin (TM) and endothelial protein C receptors (EPCR), contributing to a hypercoagulable state. Reduced TM expression leads to a downregulation of the activated protein C pathway, allowing persistence of activated cofactors factor Va and factor VIIIa, thus further promoting thrombin generation and microthrombosis (Figure 2). Increased PAI1 is a prominent feature in LC cases, leading to impaired fibrinolysis, which further promotes persistent clot formation [1]. Zuo Y et al. reported that sera from hospitalized COVID-19 patients commonly contain antiphospholipid antibodies [59], which can activate endothelial, complement, and coagulation pathways and further enhance thrombus formation [1]. Reduced ADAMTS13 activity in LC contributes to the persistence of ultra-large, prothrombotic vWF multimers. Activated platelets, complement, and autoantibodies stimulate neutrophils to release neutrophil extracellular traps (NETs), which strongly promote thrombosis by capturing platelets and vWF and activating the contact coagulation pathway [1]. Additionally, platelet glycoprotein IIb/IIIa can bind ligands containing the arginine–glycine–aspartic acid motif present in the SARS-CoV-2 spike protein, while the viral envelope protein can engage platelet Toll-like receptor-2. These interactions drive platelet aggregation initiated through the GP Ib-IX-V receptor complex binding to collagen and vWF at sites of vascular injury [60].

A systematic review and meta-analysis by Zuin M et al. demonstrated that over a mean follow-up of 8.5 months, the cumulative incidence of pulmonary embolism (PE) and deep vein thrombosis (DVT) in COVID-19 recovered patients were 1.2% and 2.3%, respectively [61]. Fan BE revealed that a subset of patients recovering from COVID-19 over a 16-month period showed raised D-dimer, factor VIII, IL-6 and von Willebrand factor (vWF) [62]. Pretorius E et al. showed that plasma samples from LC cases contain large anomalous (amyloid) deposits (microclots) which are resistant to fibrinolysis even after trypsinisation. A substantial increase in α(2)-antiplasmin (α2AP) and various fibrinogen chains, as well as Serum Amyloid A (SAA), was found trapped in the solubilized fibrinolytic-resistant pellet deposits of these cases, suggesting that lingering symptoms in LC patients might be due to the presence of these persistent circulating plasma microclots [63].

### 3.8. Mast Cell Activation

**Figure 3:**
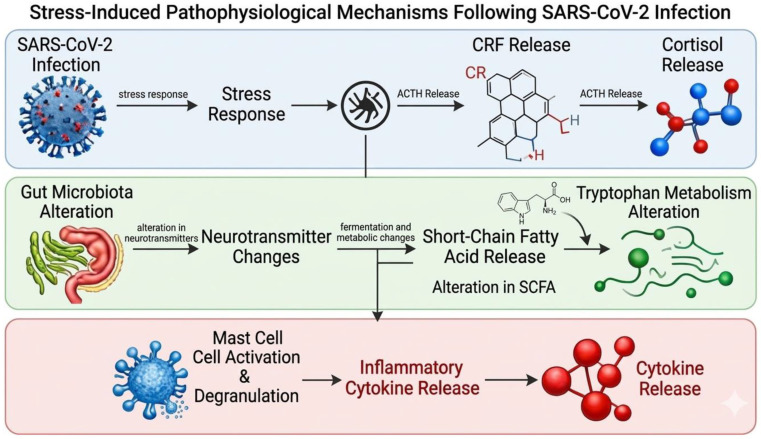
SARS-CoV-2 infection-mediated mast cell activation.

It has been observed that LC patients show abnormally increased activation of atypical mast cells (MCs) and exhibit symptoms that often mimic the symptoms and disease severity observed in individuals with idiopathic mast cell activation syndrome. This clinical overlap has led researchers to propose that persistent mast cell dysregulation may be an explanatory component in at least a subset of LC patients [64]. Mast cells are located in close proximity to nerves and their activation can influence neuroinflammatory processes by releasing histamine and cytokines that disrupt blood–brain barrier integrity and alter neuronal signaling. Proposed models suggest this could help explain neurological and cognitive symptoms frequently described in LC (e.g., brain fog, sleep disturbances) [65]. Stress induced by SARS-CoV-2 infection enhances the release of corticotropin-releasing factor (CRF), ACTH and cortisol. Concurrently, SARS-CoV-2-mediated stress leads to alterations in the gut microbiota, neurotransmitter signaling, short-chain fatty acid production, and tryptophan metabolism. Collectively, these pathophysiological changes promote mast cell activation and degranulation, resulting in the release of proinflammatory cytokines (Figure 3) [66].

COVID-19 postmortem studies have widely demonstrated hyperactivation of MCs leading to a pattern of hyperinflammation that is consistent with an inflammatory response mediated through MC activation [64,67,68,69,70]. Tan J et al. observed widespread degranulation of MCs during acute and unresolved airway inflammation in SARS-CoV-2-infected mice and non-human primates. Transcriptional changes seen in SARS-CoV-2-infected human patients requiring oxygen supplementation also implicated cells with an MC phenotype and MC activation in humans, which were confirmed through detection of the MC-specific protease, chymase, levels of which were significantly correlated with disease severity [71]. MC-derived chymase can independently generate angiotensin II from angiotensin I without the catalytic activity of ACE; hence, it is often associated with vascular diseases [72,73,74]. Interestingly, MCs express coronavirus receptor CD26 (dipeptidylpeptidase), a multi-functional type-II transmembrane glycoprotein hypothesized to contribute to SARS-CoV-2-mediated pulmonary inflammation [75].

### 3.9. Immunopathogenesis of Respiratory Consequences Associated with LC

Common respiratory complications in LC cases comprise dyspnea, fatigue, and a lingering cough, commonly occurring four weeks after diagnosis [76]. Prevalence of cough in LC cases varies in studies and meta-analyses, generally falling between 7% and 15% for non-hospitalized and hospitalized survivors, respectively, irrespective of mechanical ventilation [77,78]. LC patients have also presented evidence of high burden of incident use of bronchodilators, antitussives, expectorants, anti-asthmatics and glucocorticoids [79]. Dyspnea has been observed more frequently in individuals hospitalized at least one year after COVID-19, with lower forced expiratory volume in comparison with those without dyspnea [80].

**Figure 4:**
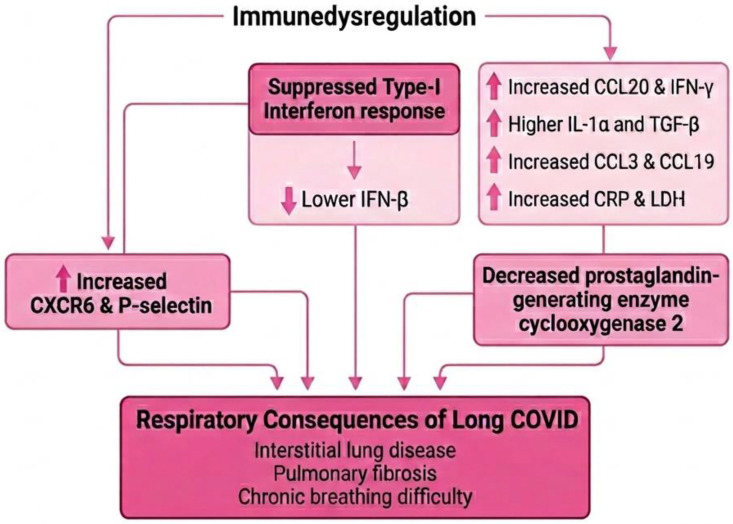
Immune dysregulation associated with respiratory sequelae of Long COVID.

Scott NA et al. reported that LC patients with persistent lung injury show increased monocyte expression of CXCR6 and the adhesion molecule P-selectin glycoprotein ligand-1, which is also present in progressive fibrosing interstitial lung disease, thus hypothetically supporting the involvement of the CXCR6–CXCL16 axis in sustained lung damage. In addition, monocytes from patients with ongoing fatigue displayed persistently reduced expression of cyclooxygenase-2 and CXCR2 [81]. Higher levels of inflammatory chemokines, including CCL20, IFN-γ, CCL3, and CCL19, have also been linked to pulmonary complications in Long COVID [82]. Colarusso C et al. showed that CRP, complement complex C5b-9, and LDH, but not IL-6, remain elevated in LC independent of disease severity or fibrosis, while increased IL-1α and TGF-β combined with reduced IFN-β predicted a higher risk of fibrotic lung changes [83]. Persistent suppression of the type-I interferon response after viral clearance may contribute to ongoing inflammation [84,85]. Furthermore, SARS-CoV-2 engagement of CD16 and ACE2 receptors can trigger inflammasome activation in macrophages, a key mechanism implicated in chronic respiratory dysfunction in LC [86,87]. Figure 4 depicts the immune dysregulation associated with respiratory sequelae of LC.

### 3.10. Immunopathogenesis of Neurological Alterations of LC

Up to one-third of individuals with LC may have ongoing neurological problems that manifest as anosmia, hypogeusia, “brain fog”, dysautonomia, cognitive impairment, peripheral neuropathy, concentration difficulty, memory loss, dizziness, and disequilibrium [88]. Mechanisms involved in SARS-CoV-2-induced central nervous system (CNS) changes are as follows:

#### 3.10.1. Direct Viral Invasion of the CNS

The route of SARS-CoV-2 entry into the CNS includes either of the following two ways:

(i) Transport along cranial and peripheral motor, sensory, or autonomic nerves (e.g., olfactory to CNS invasion), where the virus invades nerve endings and is actively transported within neurons to the brain [89].
(ii) Hematogenous spread, where virus passes from alveolar epithelial cells or endothelial cells of other organs into the blood circulation, following which the virus penetrates the blood–brain barrier (BBB), leading to neuroinvasion [89,90].

SARS-CoV-2 shows significant tropism for endothelial cells via ACE2 and TMPRSS2, enabling infection and activation of vascular endothelium across organs, including the brain [91,92]. BBB breakdown is increasingly documented in patients with LC, especially those with brain fog. Neuroimaging and biomarker studies have demonstrated persistent BBB leakage months after infection, indicating chronic neurovascular injury [93], thus causing peripheral cytokines, immune cells, and neurotoxic molecules to enter the CNS, triggering neuroinflammation and neuronal dysfunction. Once BBB integrity is compromised, circulating inflammatory mediators infiltrate the brain and activate glial cells, promoting chronic neuroinflammation and contributing to cognitive impairment, memory deficits, impaired attention and executive function [94]. Importantly, sustained systemic inflammation and abnormal immune–endothelial interactions appear to perpetuate localized BBB dysfunction even after viral clearance [91]. Brain endothelial activation in LC promotes a prothrombotic state. Activated endothelial cells upregulate adhesion molecules (ICAM-1, VCAM-1, selectins), facilitating leukocyte adhesion and platelet aggregation. Neuropathological studies have demonstrated cerebral microthrombosis, small-vessel vasculitis, and diffuse endothelial dysfunction that impair oxygen delivery and induce local hypoxia, mitochondrial dysfunction, and neuronal apoptosis [95]. Such microcirculatory impairment is strongly linked to symptoms like fatigue, cognitive dysfunction, and neuropsychiatric manifestations in LC [91]. Cerebral microvascular injury disrupts neurovascular coupling, leading to impaired cerebral perfusion and metabolic dysregulation in brain regions responsible for cognition and memory [96]. Neuroimaging studies have shown BBB breakdown and altered brain microstructure in LC patients with persistent cognitive complaints, suggesting potential overlap with mechanisms of neurodegeneration [97]. 

#### 3.10.2. Indirect Immune-Mediated Viral Effects on the CNS

Proinflammatory cytokines are released by activated peripheral immune cells in response to pathogen-associated molecular patterns (PAMPs) detected by pattern-recognition receptors (PRRs). These cytokines act on the brain via two pathways of communication [98]:

- A neural route via primary afferent neurons, including the vagus nerve, innervating the site of the infection;
- A humoral route, where peripheral cytokines or PAMPs cross the BBB at the circumventricular organs and choroid plexus and stimulate the production of proinflammatory cytokines by brain immune cells.

#### 3.10.3. Neuroinflammation and Microglial Cell Dysregulation

**Figure 5:**
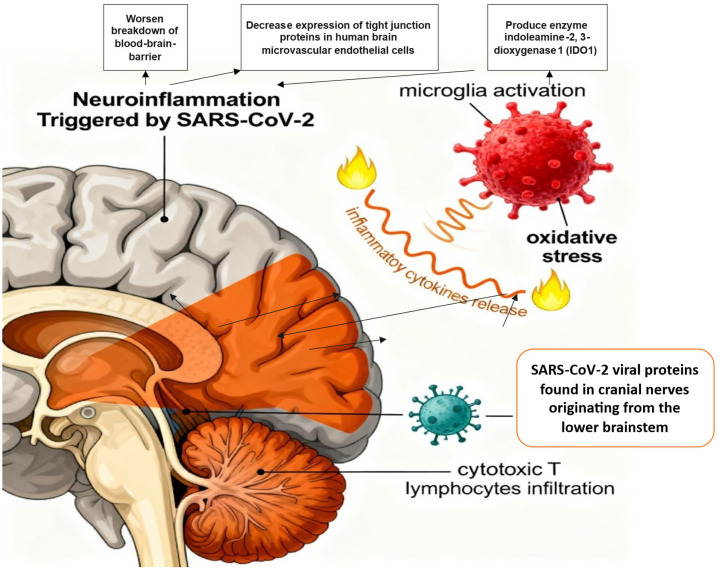
SARS-CoV-2 virus-mediated neuroinflammation and microglial cell dysregulation.

Direct or indirect viral effects on the CNS trigger neuroinflammation, resulting in long-term activation of microglia, local release of inflammatory cytokines, and oxidative stress [99,100]. A case series reported marked microglial activation and prominent infiltration of cytotoxic T lymphocytes in the brainstem and cerebellum of Long COVID patients, with meningeal T-cell infiltration observed in 79% of cases. SARS-CoV-2 viral proteins were also detected in cranial nerves arising from the lower brainstem and in scattered brainstem cells [101]. The SARS-CoV-2 spike protein has the potential to activate brain endothelial cells, leading to an inflammatory reaction that could worsen the breakdown of the BBB. Recombinant spike protein can decrease the expression of tight junction (TJ) proteins, such as ZO-1, ZO-2, Claudin-5, and junctional adhesion molecule (JAM-2), in human brain microvascular endothelial cells. It has also been speculated that SARS-CoV-2-induced neurological problems may be caused by the anti-inflammatory enzyme indoleamine-2, 3-dioxygenase 1 (IDO1), which is expressed by a variety of immune cells, including macrophages, monocytes, and microglia [102]. Figure 5 shows SARS-CoV-2 virus-mediated neuroinflammation and microglial cell dysregulation.

#### 3.10.4. Tropism of SARS-CoV-2 for Olfactory Epithelium

It has been observed that direct neurotropic actions of SARS-CoV-2 have a limited impact on the etiology of LC [103]. SARS-CoV-2 may also enter the CNS via the nasal cavity, through the foramina of the cribriform plate, and into the olfactory epithelium, where the virus exploits the close vicinity of olfactory sensory neurons whose axons project into the olfactory bulb of the brain [104]. De Melo GD et al. showed that analysis of olfactory mucosa from individuals with persistent COVID-19-related anosmia detected viral transcripts and SARS-CoV-2-infected cells alongside sustained inflammation [105]. Another investigation identified SARS-CoV-2 RNA in 53% of examined COVID-19 autopsy brain samples [101]. In a separate study of 18 COVID-19 autopsies, Lee MH et al. reported macrophage infiltration and activation of astrocytes and microglia within perivascular spaces of the olfactory bulb and brainstem in 13 cases, while ultra-high-resolution 11.7-Tesla imaging revealed punctate hyperintensities in 9 of 13 brains, consistent with microvascular injury and fibrinogen leakage [106].

### 3.11. Immunopathogenesis of Gastroenterological Involvement (GI) in LC

GI manifestations in LC cases are believed to be due to the ACE2-expressing cells on the brush border of the small intestinal mucosa [107]. Previous studies have reported that SARS-CoV-1 and Middle East respiratory syndrome coronavirus (MERS-CoV) RNA were detected in stool samples, and these viruses showed tropism for intestinal epithelial cells [108,109]. The structural similarity between the spike proteins of SARS-CoV-1 and SARS-CoV-2 and the presence of ACE2 in intestinal epithelial cells support the hypothesis of direct viral infection followed by an immune-driven inflammatory response, contributing to GI manifestations [110].

**Figure 6:**
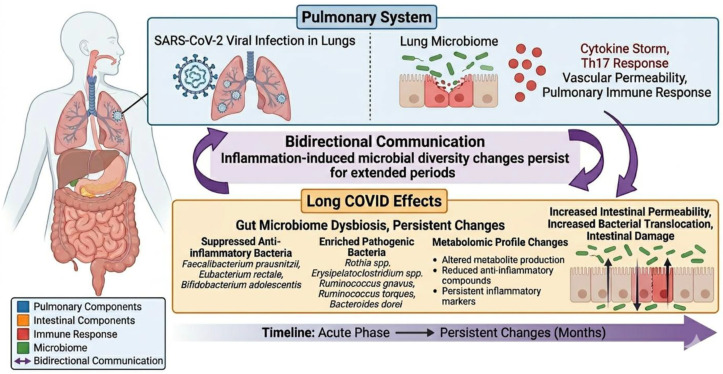
Gut–lung axis in SARS-CoV-2 infection and Long COVID.

Bidirectional communication exists between the intestine and lung, which explains the fact that an intact intestinal barrier modulates pulmonary immune responses and the lung microbiome. In cases of SARS-CoV-2 infection, intestinal changes may be triggered due to the impact of the virus on intestinal permeability and promoting bacterial translocation [111]. The acute exacerbated immune responses triggered by SARS-CoV-2 have been linked to a Th17 immune response, which leads to vascular permeability and leakage, resembling the immune response seen in cases of bacterial translocation [112]. This evidence suggests that the cytokine storm induced by SARS-CoV-2 might be responsible for intestinal damage seen in LC [110]. Another study showed that changes in microbial diversity induced by inflammation during SARS-CoV-2 infection can persist for months, leading to alterations in the metabolomic profile that involve the suppression of anti-inflammatory bacteria like *Faecalibacterium prausnitzii*, *Eubacterium rectale*, and *Bifidobacterium adolescentis*, and the enrichment of pathogens, including *Rothia* spp., *Erysipelatoclostridium* spp., *Ruminococcus gnavus*, *Ruminococcus torques*, and *Bacteroides dorei* [113,114]. The gut–lung axis in SARS-CoV-2 infection and LC is shown in Figure 6. Brogna C et al. demonstrated that SARS-CoV-2 could also infect bacteria in the gut microbiota, indicating that SARS-CoV-2 could act as a bacteriophage, which can lead to unique viral persistence and contribute to intestinal dysbiosis in LC [115].

### 3.12. Immunopathogenesis of Cardiovascular System Involvement (CVS) in LC

While the exact cause behind enduring cardiac injury in LC cases is not yet fully understood, yet a potential scenario points to a persistent inflammatory reaction reflected by the presence of circulating biomarkers for 3 to 8 months after acute infection: CRP, IL 6 and 8, ferritin, IFN beta and gamma1, procalcitonin, chemokine ligand (CXCL) 9, CXCL10, T-cell immunoglobulin mucin-3 (TIM-3), plasma ACE2 activity, and pentraxin 3 (PTX3) (activates complement and facilitates pathogen recognition by macrophages) [27].

SARS-CoV-2 resides within the interstitial cells and infiltrating macrophages within the myocardium. Viral entry depends upon the interaction between the viral spike glycoprotein, the ACE2 receptor, and the host cell protease system, such as TMPRSS2. ACE2 plays a crucial role in the neurohumoral regulation of the CVS and is primarily present in the vascular endothelium, cardiomyocytes, pericytes, cardiac fibroblasts, and epicardial adipose tissue. ACE2 transforms angiotensin 1 and 2 into peptides with cardioprotective properties. Downregulation of ACE2 by SARS-CoV-2 prevents the synthesis of cardioprotective peptides, thus predisposing to cardiovascular damage [116].

Another proposed mechanism of myocardial damage is mediated by reactive oxygen species, which can induce the release of internal histones, damage-associated molecular patterns (DAMPs), and oxidized lipid–protein complexes that trigger an inflammatory response resulting in myocardial tissue injury and chronic myocardial scarring. This scarring may lead to reduced ventricular compliance, impaired blood flow within the heart muscle, decreased cardiac muscle contraction, and irregular heartbeats. Cytokine-induced damage can also lead to blood clot formation, reduced oxygen delivery, destabilization of coronary plaques, transition of chronic heart conditions into unstable states, increased metabolic requirements, diminished cardiac capacity, and inflammation in the heart valves [27].

### 3.13. Immunopathogenesis of Renal Involvement in LC

Renal involvement in LC patients manifests as hematuria, proteinuria, acute kidney injury (AKI) and end-stage kidney disease (ESKD) [117,118,119]. Earlier studies indicate that SARS-CoV-2 can utilize the transmembrane glycoprotein CD147, which is abundantly expressed on proximal tubular epithelial cells, and this pathway has been implicated in immune-inflammatory injury and cell-cycle dysregulation in renal disease [120,121]. In an autopsy series of 26 COVID-19 patients, Su H et al. described widespread proximal tubular damage characterized by brush border loss, vacuolar degeneration, and areas of necrosis on light microscopy. Electron microscopy revealed clusters of coronavirus-like particles within tubular epithelium and podocytes, and immunostaining for SARS-CoV nucleoprotein was positive in tubules, providing direct evidence of viral invasion of kidney tissue [119]. Endothelial damage and microvascular dysfunction lead to ongoing vascular permeability and microvascular thrombosis, limiting oxygen delivery and causing chronic ischemia, which may impair renal perfusion and contribute to chronic injury in LC cases. While evidence is emerging, this mechanism is supported by systemic vascular studies in post-COVID-19 syndromes [122]. LC is associated with terminal complement system dysregulation, including continuous activation of alternative and classical pathways, thus increasing renal injury, thrombosis, and promoting pro-fibrotic processes [122].

**Figure 7:**
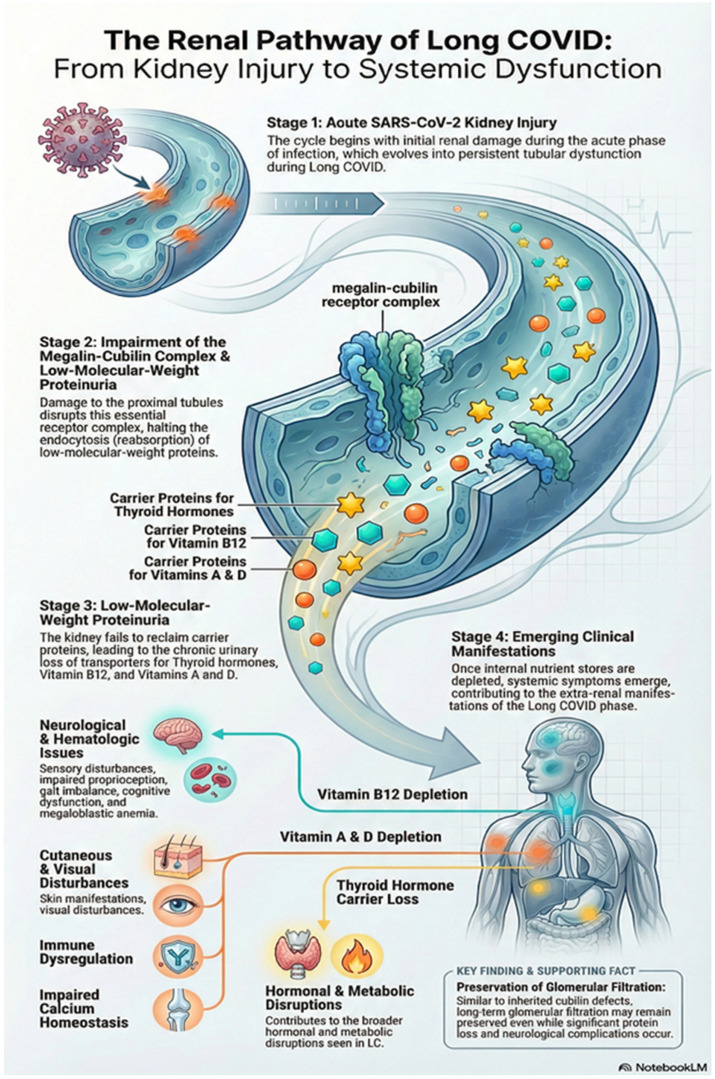
Megalin–cubilin receptor complex mutation leading to Long COVID manifestations.

Evidence from inherited loss-of-function mutations affecting proximal tubular endocytosis demonstrates that disruption of renal protein reabsorption can result in significant downstream pathology. A well-recognized example involves mutations in megalin and cubilin, which together constitute the principal receptor complex mediating proximal tubular endocytosis. Dysfunction of this system leads to low-molecular-weight proteinuria, including urinary loss of carrier proteins for thyroid hormones, vitamin B12, and the fat-soluble vitamins A and D. In the context of LC, persistent tubular dysfunction following acute SARS-CoV-2-associated kidney injury may similarly impair megalin–cubilin-mediated reabsorption. Although body stores of these vitamins typically delay the onset of symptomatic deficiency, sustained urinary losses over time may result in clinical manifestations emerging months after the acute infection, thereby contributing to the LC phase. Individuals with pre-existing nutritional compromise may experience earlier symptom onset. Deficiencies of vitamins A and D can lead to cutaneous manifestations, visual disturbances, immune dysregulation, and impaired calcium homeostasis. Vitamin B12 deficiency may result in sensory disturbances, impaired proprioception, gait imbalance, cognitive dysfunction, and megaloblastic anemia. Notably, in inherited cubilin defects, despite persistent proteinuria, long-term glomerular filtration is often preserved; however, affected individuals remain at significant risk of neurological and hematologic complications. These observations raise the possibility that chronic tubular protein loss following COVID-19-related renal injury may contribute to some of the extra-renal manifestations observed in LC [123]. Figure 7 shows the megalin–cubilin receptor complex mutation leading to LC manifestations. Immune-mediated inflammation and tubular injury during SARS-CoV-2 infection can impair the reabsorption of electrolytes, amino acids, and nutrients, leading to altered tryptophan metabolism that further causes neurocognitive symptoms evident in LC [123].

### 3.14. Immunopathogenesis of Dermatological Involvement in LC

**Figure 8:**
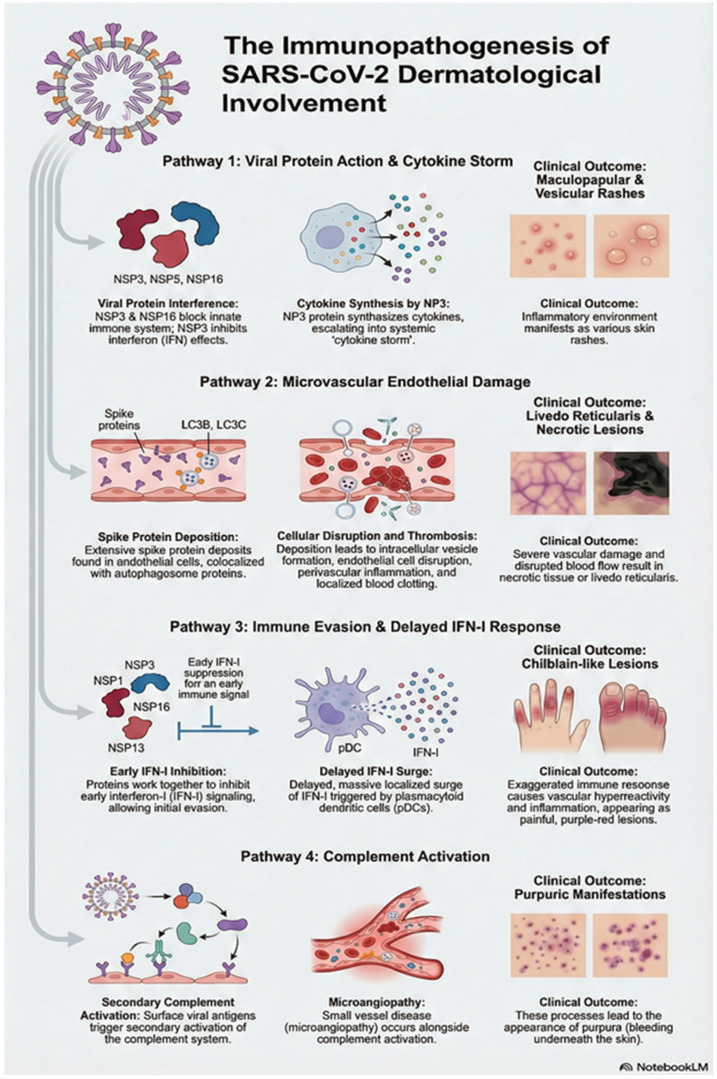
Immunopathogenesis of dermatological involvement in Long COVID.

Skin manifestations described in conjunction with COVID-19 include maculopapular rash, urticaria, petechiae, purpura, vesicles, chilblains, livedo racemose, and ischemia of the distal segments of the limbs [124,125]. Magro C. et al. hypothesized that interstitial and perivascular neutrophilia with pronounced leukocytoclasia and complement protein deposition in the dermal capillaries point to vasculitis phenomena [126]. Sanchez A. et al. claimed that the rash occurs as a direct effect of the virus, justified by the presence of lymphocytosis (without eosinophils), papillary dermal edema, epidermal spongiosis, and lymph histiocytic infiltrates [125]. Although the pathophysiological mechanisms of skin lesions in LC patients is still hypothetical; it has been assumed that the possible actions of SARS-CoV-2 on the skin may be mediated by non-structural viral proteins (NSPs) that block the innate immune system (NSP3, NSP16), inhibit the effect of IFN (NSP5), or synthesize cytokines (NP3), which further can lead to cytokine storm, thus leading to the appearance of various maculopapular or vesicular rashes. Studies have shown vasculitic skin lesions induced by SARS-CoV-2 virus are caused by extensive spike protein depositions in microvascular endothelial cells colocalized with the autophagosome proteins LC3B and LC3C, thus leading to intracellular vesicle formation, endothelial cell disruption, perivascular inflammation, and thrombosis, and resulting in livedo reticularis or necrotic lesions [127]. The immune evasion mechanisms of various NSPs (such as NSP1, NSP3, NSP13, and NSP16) that inhibit early interferon-I (IFN-I) signaling can lead to a delayed, massive, and localized surge of IFN-I. This exaggerated immune response (mediated by plasmacytoid dendritic cells) leads to vascular hyperreactivity and inflammation, manifesting as chilblain-like lesions [128]. Also, secondary activation of complement by the surface viral antigen and microangiopathy may lead to the appearance of purpuric manifestations [129,130]. Immunopathogenesis of dermatological involvement in LC is shown in Figure 8. Another possible effect of SARS-CoV-2 is a direct impact through ACE2 in the epidermis, causing acantholysis and dyskeratosis [131] as ACE2 appears in the basal epidermal layer, dermal blood vessel endothelial cells and eccrine tissue [39,129].

### 3.15. Immunopathogenesis of Myalgic Encephalomyelitis and Chronic Fatigue Syndrome (ME/CFS) in LC

**Figure 9:**
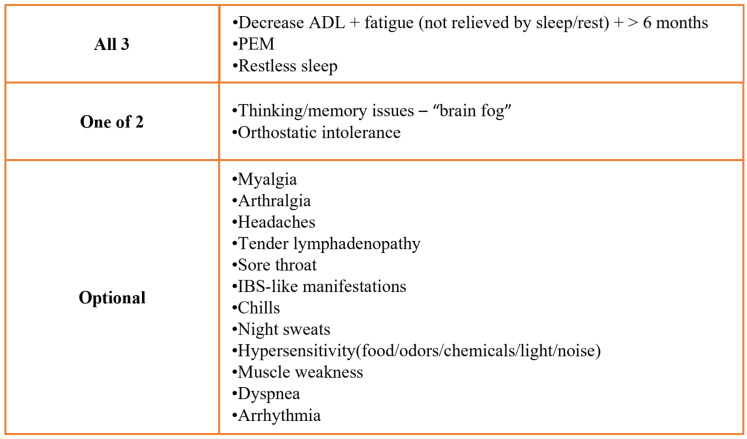
ME/CFS clinical picture.

ME/CFS is clinically supported by the presence of a polymorphic clinical picture, as shown in Figure 9. There exists clear symptom overlap of ME/CFS with LC, encompassing the key features of post-exertional fatigue, neurocognitive symptoms, dysautonomia and postural orthostatic tachycardic syndrome [1]. A systematic review found that 25 of 29 ME/CFS symptoms were reported by at least one selected LC study [132], whereas another study found common hub proteins, such as IL-6 and IL-1β, between the two conditions [133]. Interestingly, even the immunopathological mechanisms of the two conditions exhibit overlap. It is noteworthy that raised CCL11, which has credentials as an LC biomarker and is functionally linked to neurocognitive symptoms, is also a biomarker of ME/CFS [134].

ADL—Activity of daily living; PEM—Post-exertional malaise; IBS—Irritable bowel syndrome; and ME/CFS—Myalgic encephalomyelitis/chronic fatigue syndrome.

### 3.16. Immunopathogenesis of Mild Constitutional Symptoms

Almost all LC patients exhibit constitutional symptoms of fever, chills, headache, arthralgia, and mild dyspnea, which can be attributed to the release of proinflammatory cytokines, most of which are pyrogenic (IL-6, IL-1, IFN-γ, and TNF-α). In LC patients with diabetic neuropathy, neuropathic pain correlated with increased levels of IL-6 and IL-10 for large nerve fiber damage [135], and patients with polyneuropathies show elevated serum cytokines IL-8 and TNF-α and lower expression of IL-10 [136].

Cytokines cause serotonin release in the brain, producing extreme central fatigue [95]. In healthy individuals, administration of IL-6 and IL-1β produces fatigue, sleep disturbances, and difficulty in concentration [137].

Post-exertional malaise (PEM), or post-exertional symptom exacerbation (PESE), one of the most striking and demoralizing symptoms of LC, is characterized by worsening or relapse of symptoms such as extreme fatigue, malaise, fever, breathlessness, and neurological deficits after physical or mental activity [3]. It has been evaluated in previous studies that modest increases in IL-6 are expected after exercise of low-to-moderate intensity [138]. Low RN et al. claimed that exercise-induced cytokines may trigger a recurrence of symptoms by reinitiating the same feedback loop pathways that caused the initial symptoms of COVID-19 [29].

## 4. Summary

LC is characterized by sustained immune dysregulation even after the acute infection resolves. emphasizing persistent activation and exhaustion of both T and B lymphocytes, suggesting that prolonged immune stimulation and impaired antiviral responses may drive ongoing symptoms. Current evidence supports viral persistence as a potential driver of LC, but uncertainty persists regarding whether detected viral material represents replication-competent virus or residual antigens, highlighting key methodological limitations. Hypothesis prevails that T- and B-cell exhaustion, along with viral persistence, leads to failure of viral clearance culminating in LC manifestations.

EBV reactivation is frequently reported in LC and severe COVID-19 and correlates with inflammation, immune dysfunction, prolonged ICU stay, and worse outcomes. Proposed mechanisms suggest a bidirectional interaction in which SARS-CoV-2-induced inflammation promotes EBV reactivation, while EBV may enhance ACE2 expression and viral entry, potentially amplifying disease severity. However, current evidence is largely associative, with major heterogeneity and confounding from disease severity and treatments.

COVID-19 is associated with increased autoantibody production, including those linked to SLE, inflammatory myopathies, and receptors such as ACE and muscarinic receptors, with some correlations to LC symptom severity. Proposed mechanisms include molecular mimicry, cytokine-driven inflammation, and epigenetic changes that may sustain chronic inflammation and neuroimmune dysfunction. However, findings remain inconsistent, particularly regarding anti-interferon antibodies and their persistence after the acute phase. Overall, autoimmunity is a plausible contributor to LC, but heterogeneity and conflicting evidence limit definitive causal conclusions.

LC is linked to dysregulated antiviral immunity during acute infection, particularly delayed or impaired interferon responses that may permit higher viral burden, persistence in immune-privileged tissues, and chronic inflammation. However, studies of antibody and T-cell responses to SARS-CoV-2 antigens show conflicting results, reporting both reduced and exaggerated immune responses in different cohorts. These inconsistencies suggest substantial heterogeneity in immune trajectories and highlight uncertainty about whether immune deficits or excessive activation primarily drive LC. Overall, evidence supports a role for abnormal early antiviral responses, but causal mechanisms remain unresolved.

Persistent endothelial injury appears central to LC-associated immunothrombosis, with sustained procoagulant signaling, impaired fibrinolysis, and reduced anticoagulant pathways reinforcing a chronic hypercoagulable state. The integration of autoantibodies, platelet hyperactivation, NET formation, and reduced ADAMTS13 activity suggests a self-perpetuating thromboinflammatory loop rather than a transient post-infection effect. However, many mechanisms are inferred from acute COVID-19 data, highlighting a key limitation and the need for longitudinal studies to confirm causality in Long COVID. Overall, the evidence supports endothelial dysfunction as a unifying but still incompletely defined driver of persistent microvascular pathology.

Persistent mast cell dysregulation may contribute to LC in a subset of patients, linking immune, neuroinflammatory, and stress-related pathways to symptoms such as brain fog and sleep disturbance. Mechanistic models propose that infection-driven neuroendocrine and gut–brain axis changes sustain mast cell activation and cytokine release, reinforcing chronic inflammation. However, much of the supporting evidence comes from acute infection, animal models, and indirect biomarkers, limiting causal inference in Long COVID. Overall, mast cell activation is a plausible but still incompletely validated mechanism requiring targeted longitudinal and interventional studies.

Persistent respiratory symptoms in LC appear linked to ongoing immune dysregulation rather than residual viral infection alone, with chemokine-driven monocyte recruitment and suppressed type-I interferon responses sustaining lung inflammation. Biomarker patterns suggest a shift toward fibrotic and complement-mediated injury pathways, highlighting a risk of progressive interstitial lung disease-like changes. However, variability in prevalence and biomarker findings across studies underscores heterogeneity in patient populations and limits clear causal conclusions. Overall, the evidence supports chronic immune-mediated lung injury as a key driver of long-term respiratory sequelae, but standardized longitudinal studies are needed to define mechanisms and predictors.

Neurological LC likely reflects a multifactorial process involving blood–brain barrier disruption, chronic neuroinflammation, and microvascular injury rather than direct viral neuroinvasion alone. Evidence supports persistent immune–endothelial dysfunction, microthrombosis, and microglial activation as key drivers of cognitive and neuropsychiatric symptoms. However, many findings are derived from small cohorts, autopsy studies, or indirect biomarkers, limiting causal inference and generalizability. Overall, the data suggest overlapping pathways with neurodegenerative and vascular disorders, but longitudinal and mechanistic studies are needed to define their relative contributions.

Gastrointestinal LC likely reflects a combination of direct viral effects, immune-driven inflammation, and persistent gut dysbiosis mediated through ACE2-rich intestinal epithelium and the gut–lung axis. Evidence linking microbial imbalance, Th17 responses, and increased intestinal permeability suggests ongoing immune activation and bacterial translocation may sustain symptoms. However, several mechanisms remain speculative, particularly viral persistence and bacteriophage-like behavior, highlighting limited direct human evidence. Overall, persistent microbiome and barrier dysfunction emerge as key hypotheses requiring longitudinal and mechanistic validation.

Cardiovascular LC appears driven by persistent inflammation, ACE2 dysregulation, and oxidative stress that together promote myocardial injury, fibrosis, and thromboinflammatory complications. Sustained elevation of inflammatory and complement-related biomarkers supports ongoing immune activation months after infection. However, direct viral persistence in cardiac tissue and the relative contribution of pre-existing cardiovascular risk remain unclear. Overall, current evidence suggests multifactorial chronic cardiac injury but highlights the need for longitudinal mechanistic and imaging studies.

Renal LC appears to involve direct viral tropism, endothelial injury, complement activation, and microvascular thrombosis, collectively promoting chronic ischemia and fibrosis. The proposed link between persistent tubular dysfunction and long-term nutrient and protein losses offers a novel explanation for some extra-renal and neurological symptoms. However, much of the evidence relies on acute-phase autopsy data and extrapolation from genetic disorders, limiting direct proof in LC. Overall, chronic tubular and microvascular injury is a compelling but still evolving mechanism requiring longitudinal clinical validation.

Dermatological LC manifestations likely reflect immune-mediated vascular and inflammatory processes, including complement activation, microangiopathy, and delayed interferon responses leading to vasculitic and chilblain-like lesions. Proposed roles for viral proteins and ACE2 expression in skin suggest both direct and indirect mechanisms of injury. However, most mechanisms remain hypothetical and extrapolated from acute infection or small studies. Overall, cutaneous findings appear to mirror systemic immune–vascular dysfunction but require targeted longitudinal research for confirmation.

LC shows substantial clinical and immunological overlap with ME/CFS, particularly in post-exertional fatigue, dysautonomia, and neurocognitive dysfunction, suggesting shared pathophysiological pathways. Common inflammatory mediators and biomarkers (e.g., IL-6, IL-1β, CCL11) support the hypothesis of convergent immune dysregulation. However, current evidence is largely associative and does not establish whether LC represents a trigger, subtype, or distinct entity within ME/CFS. Overall, the overlap is compelling but requires mechanistic and longitudinal studies to clarify causality and disease boundaries.

Mild constitutional LC symptoms appear driven by persistent low-grade cytokine activity that promotes fatigue, pain, and neurocognitive disturbances, with post-exertional malaise reflecting abnormal immune responses to physical or mental stress. Evidence linking exercise-induced cytokines to symptom relapse supports a self-reinforcing inflammatory feedback loop. However, most findings are associative and extrapolated from experimental cytokine studies rather than LC-specific trials. Overall, chronic immune activation provides a plausible framework but requires longitudinal mechanistic validation.

Table 1 describes the association of organ-specific immune dysregulation with LC manifestations.

## 5. Conclusions

The pathophysiology of LC is complex and encompasses wide variations as per different studies, along with a lack of consistent and robust evidence of causal association between the numerous manifestations and their respective pathogenesis. Current evidence suggests that its immunopathogenesis is driven by a dynamic interplay between persistent immune activation, viral antigen persistence, immune dysregulation, autoimmunity, endothelial dysfunction, microvascular injury, and aberrant tissue repair mechanisms. However, significant heterogeneity exists across studies in terms of patient populations, clinical phenotyping, duration of follow-up, and methodological approaches, leading to variability in reported findings.

Although numerous studies have explored individual components of LC, like chronic inflammation, T-cell exhaustion, B-cell dysregulation, autoantibody production, cytokine imbalances, dysautonomia, and organ-specific immune injury, the causal relationships between these immunological abnormalities and specific clinical manifestations remain incompletely defined. It remains unclear whether these immune alterations represent persistent viral activity, post-infectious immune imprinting, bystander immune activation, reactivation of latent pathogens, or secondary consequences of tissue damage.

Importantly, most available data are derived from cross-sectional or small-cohort studies, limiting the ability to establish temporal and mechanistic associations. There is also a lack of standardized diagnostic criteria, validated biomarkers, and uniform immunological profiling strategies. This has hindered the development of clear immunopathogenic models and targeted therapeutic approaches.

Future research must prioritize longitudinal, multicenter studies with standardized case definitions and integrated approaches, including immunophenotyping, transcriptomics, proteomics, metabolomics, and virological assessment, to delineate causal pathways. Stratification of patients based on immunological endotypes may help identify distinct pathogenic mechanisms underlying different clinical phenotypes of LC. Such structured and comprehensive investigations are essential to move beyond associative findings toward mechanistic clarity.

A deeper understanding of the immunopathogenesis of LC will not only clarify disease mechanisms but also facilitate biomarker discovery, risk stratification, and development of precision-based immunomodulatory therapies. Until then, LC remains a syndrome characterized by biological complexity, clinical heterogeneity, and substantial gaps in mechanistic insight.

## Figures and Tables

**Table 1:** Association of organ-specific immune dysregulation with Long COVID manifestations.

S.No.	Organ	LC Manifestations	Immune Dysregulation
1.	Endothelium	Microvascular ischemia Venous and arterial thromboembolism Inflammation across multiple organs	Endothelial upregulation of P-selectin, TF, ICAM-1, VCAM-1, PAI-1 and release of ultra-large vWF multimers, along with reduced thrombomodulin and EPCR, promotes thrombin generation and microthrombosis formation [1]. Elevated PAI-1 in LC impairs fibrinolysis and promotes persistent clot formation [1,59]. Activated platelets, complement and autoantibodies trigger neutrophil extracellular trap (NET) formation, which captures platelets/vWF and promotes platelet aggregation [1,60]
2.	Mast cells	Neuroinflammation caused by mast cell mediators that disrupt the blood–brain barrier and neuronal signaling may underlie cognitive dysfunction, “brain fog,” and sleep disturbances commonly reported in LC [65]. Chronic mast cell-driven cytokine release and hyperinflammation contribute to persistent airway inflammation and respiratory symptoms [64,67,68,69,70,71,75]. Mast cell-derived chymase-mediated angiotensin II generation promotes vascular dysfunction, potentially contributing to cardiovascular and systemic inflammatory manifestations in LC [72,73,74].	SARS-CoV-2-related stress responses (CRF, ACTH, cortisol), gut microbiota alterations, neurotransmitter imbalance, and disrupted tryptophan/SCFA metabolism promote mast cell degranulation [66] Expression of CD26 on mast cells contributes to mast cell hyperactivation and pulmonary inflammation [64,67,68,69,70,71,72,73,74,75]
3.	Respiratory System	Persistent inflammation and fibrotic signaling pathways contribute to reduced lung function and long-term dyspnea in LC patients [80,83]. Continued immune cell recruitment and chemokine elevation drive chronic airway inflammation, explaining lingering cough and increased use of respiratory medications [77,78,79,80,81,82]. Ongoing inflammasome activation and impaired antiviral responses sustain chronic respiratory dysfunction and fatigue in LC [84,85,86,87].	Increased inflammatory chemokines (CCL20, IFN-γ, CCL3, CCL19), elevated CRP, complement C5b-9, and LDH, and reduced IFN-β indicate ongoing pulmonary inflammation and risk of fibrotic lung remodeling even after viral clearance [82,83]. Altered monocyte phenotype (↑CXCR6, ↑P-selectin glycoprotein ligand-1, ↓COX-2 and CXCR2) suggests impaired resolution of inflammation in the lungs [81]. Suppressed type-I interferon signaling and inflammasome activation via ACE2/CD16 engagement in macrophages contribute to chronic inflammatory signaling and respiratory immune dysregulation [84,85,86,87].
4.	Central Nervous System	BBB breakdown and chronic neuroinflammation lead to cognitive impairment, memory deficits, impaired attention, and “brain fog” [93,94,97]. Cerebral microvascular injury and impaired neurovascular coupling contribute to fatigue, dizziness, and neuropsychiatric symptoms due to reduced cerebral perfusion and metabolic dysregulation [91,96]. Persistent inflammation of the olfactory epithelium and CNS immune activation contribute to anosmia, dysautonomia, and other neurological symptoms observed in LC [105,106].	Sustained systemic inflammation and immune–endothelial interactions drive endothelial activation, leukocyte adhesion, platelet aggregation, and cerebral microthrombosis, resulting in chronic neuroinflammation and hypoxic neuronal injury [91,94,96]. Persistent neuroinflammation is marked by microglial activation, cytotoxic T-cell infiltration, spike-protein-induced loss of tight junction proteins, and cytokine-mediated signaling through neural and humoral pathways [98,99,100,101,102].
5.	Gastrointestinal System	Chronic intestinal inflammation and barrier disruption promote symptoms such as diarrhea, abdominal pain, and malabsorption due to ongoing epithelial injury and microbial imbalance [110,111,112,113,114]. Dysbiosis and reduced anti-inflammatory microbiota sustain systemic immune activation and metabolic disturbances, prolonging GI and extra-intestinal symptoms in LC [113,114].	Th17-mediated cytokine responses cause vascular leakage, promoting intestinal permeability and bacterial translocation and leading to intestinal damage resembling cytokine storm-related injury [110,111,112]. Persistent inflammation leads to long-term gut dysbiosis and altered metabolomic profiles, characterized by depletion of anti-inflammatory bacteria and enrichment of pathogenic species, possibly sustained by viral persistence within gut microbiota [113,114,115].
6.	Urinary System	Chronic immune-mediated tubular and vascular injury contributes to hematuria, proteinuria, AKI, and progression to ESKD in LC patients [117,118,119,120,121,122]. Persistent tubular protein loss leads to deficiencies of vitamins A, D, and B12, contributing to extra-renal manifestations such as neurological, hematologic, cutaneous, and immune dysfunction in LC [123]. Impaired electrolyte and nutrient reabsorption with altered tryptophan metabolism may contribute to neurocognitive symptoms and prolonged systemic manifestations seen in LC [123].	CD147 causes tubular cell-cycle dysregulation and proximal tubular damage, supported by histopathological evidence of viral particles in tubular epithelium and podocytes [119,120,121]. Immune-mediated tubular dysfunction impairs megalin–cubilin-mediated protein reabsorption, leading to urinary loss of vitamins and nutrients and altered tryptophan metabolism [123].
7.	Integumentary System	Immune-mediated vasculitis, microthrombosis, and complement activation contribute to rashes, purpura, petechiae, livedo reticularis, and necrotic/ischemic lesions [124,125,126,127,128,129,130]. Delayed and exaggerated interferon responses and cytokine-driven inflammation lead to persistent urticaria, maculopapular eruptions, and chilblain-like lesions in LC [125,128].	Viral immune-evasion proteins inhibit early IFN-I signaling and later provoke an exaggerated interferon-mediated inflammatory response, leading to cytokine storm-related skin inflammation and chilblain-like lesions [128]. Spike protein deposition in dermal microvasculature promotes endothelial disruption, thrombosis, complement activation, and microangiopathy, while ACE2 expression in epidermal and vascular cells allows direct viral injury to skin tissue [127,129,130,131].
8.	Cerebrovascular System	Chronic inflammation and myocardial scarring can reduce ventricular compliance, impair cardiac contraction, and promote arrhythmias, explaining LC symptoms like palpitations and exercise intolerance [27]. Cytokine-induced endothelial activation and inflammation may promote thrombosis, destabilize coronary plaques, and increase metabolic demand, contributing to angina or progression of chronic heart disease [27].	Long-term cardiac injury is linked to a sustained inflammatory state, evidenced by the presence of circulating biomarkers (such as CRP, IL-6, IL-8, and IFN-gamma) that remain elevated for 3 to 8 months after the initial infection [27]. Release of damage-associated molecular patterns (DAMPs) and oxidized lipid–protein complexes promotes myocardial tissue inflammation, contributing to chronic scarring and structural alterations [27].

TF—Tissue Factor; ICAM-1—Intercellular Adhesion Molecule-1; VCAM-1—Vascular Cell Adhesion Molecule-1; PAI-1—Plasminogen Activator Inhibitor-1; vWF—von Willebrand Factor; EPCR—Endothelial Protein C Receptor; CRF—Corticotropin-Releasing Factor; ACTH—Adrenocorticotropic Hormone; AKI—Acute Kidney Injury; ESKD—End-stage kidney disease; ↑—increased expression; ↓—decreased expression.

## Data Availability

No new data were created or analyzed in this study.
